# High risk of elevated metal concentrations with 9/10-mm stem trunnions and highly cross-linked polyethylene grafted with poly(2-methacryloyloxyethyl phosphorylcholine) in total hip arthroplasty

**DOI:** 10.1186/s13018-023-03510-4

**Published:** 2023-01-10

**Authors:** Kyosuke Kobayashi, Kenichi Kidera, Kazuteru Shiraishi, Narihiro Okazaki, Ko Chiba, Akihiko Yonekura, Makoto Osaki

**Affiliations:** Nagasaki University Hospital, 1-7-1 Sakamoto, Nagasaki City, Nagasaki 852-8501 Japan

**Keywords:** Trunnion, Fretting and corrosion, Serum metal concentrations, Adverse local tissue reaction, Metal-on-polyethylene

## Abstract

**Background:**

The risks of metal release due to fretting and corrosion at the head–neck junction and consequent adverse local tissue reaction (ALTR) have concerns in metal-on-polyethylene (MoP) total hip arthroplasty (THA). Although trunnions have become thinner in diameter to increase the range of motion, it has remained unclear whether this change affects metal release and ALTR in vivo. This study aimed to investigate serum metal concentrations and the prevalence of ALTR in MoP THA with a 9/10-mm stem trunnion.

**Patients and methods:**

A consecutive series of 37 hips that underwent THA using MoP grafted with poly(2-methacryloyloxyethyl phosphorylcholine) (PMPC) with a 9/10-mm trunnion stem were retrospectively reviewed. Serum metal levels were assessed and compared with those in MoP THA with a 10/12-mm trunnion stem. ALTR was diagnosed with serum metal levels and cross-sectional images. The factors associated with serum metal levels were also assessed.

**Results:**

The median serum cobalt and chromium levels were 1.5 μg/L and 1.0 μg/L in the 9/10-mm group and 0.2 μg/L and 0.4 μg/L in the 10/12-mm group, respectively. ALTR was found in 5 hips of 3 patients. Revision surgery was performed in 4 hips, and all stem trunnions and femoral heads showed severe corrosion. Postoperative walking ability was associated with serum metal levels.

**Conclusion:**

It was found that a 9/10-mm stem trunnion with MoP grafted with PMPC had high risks of metal release in primary THA. Careful follow-up and cross-sectional imaging are needed to detect ALTR for early revision.

## Introduction

The risks of metal release due to fretting and corrosion at the head–neck junction and consequent adverse local tissue reaction (ALTR) have concerns in metal-on-polyethylene (MoP) THA in recent years. The mechanical disruption of the passive metal oxide layer secondary to micromotion and electrochemical reactions at the head–neck junction leads to corrosion and metal release [1]. This process has potential risks of ALTR, including persistent pain, osteolysis, pseudotumor formation, necrosis of the soft tissues, and instability of the hip [2–4].

Trunnions have become shorter in length and thinner in diameter to avoid implant impingement and improve the range of motion in modern THA. However, these changes decreased the flexural rigidity of the trunnion and the contact area of the head–neck junction and increased edge loading and contact stress. Trunnion design was believed to be one of the risk factors for fretting and corrosion [5]. Although some retrieval studies reported that the fretting scores and taper wear rate were higher with a shorter and thinner trunnion stem [6, 7], it remained controversial [8–10] and unclear in vivo. Therefore, the aim of this study was to investigate serum metal concentrations, the number of ALTRs, and the factors associated with serum cobalt (Co) and chromium (Cr) levels in MoP THA with a thinner trunnion. The factors associated with serum metal levels were also assessed. The hypothesis was that serum metal levels and ALTR increase with a thinner trunnion compared to thicker MoP THAs or previous reports.

## Patients and methods

The medical records of 38 patients (43 hips) who underwent primary THA using the J-Taper prostheses (KYOCERA, Kyoto, Japan) operated under the care of five senior surgeons between September 2012 and March 2014 were retrospectively reviewed. During this period, the surgeons performed 173 THAs, of which only 43 hips met the criteria to receive the J-Taper stem, notably adequate bone quality, weight < 90 kg, and absence of severe developmental dysplasia of the hip (Crowe grade I) or greater posterior tilt of the pelvis. One patient died, four patients were lost to follow-up, and one patient refused to participate in this study. Therefore, 37 hips of 32 patients (male, *n* = 5; female, *n* = 27; mean age at surgery, 58 (range 37–74) years; follow-up, 8 (range 7–9) years) were assessed (Table 1). The underlying diagnoses were primary osteoarthritis (*n* = 4), secondary osteoarthritis due to developmental dysplasia of the hip (*n* = 24), secondary osteoarthritis due to trauma (*n* = 1), secondary osteoarthritis due to osteochondritis dissecans of the hip (*n* = 1), and osteonecrosis of the femoral head (*n* = 7).

**Table 1:** Patients’ demographic and surgical data (*n* = 32 patients, 37 hips and 6 patients, 7 hips in each group)

	J-Taper group		Synergy group		
	Median *n* (%)	Range	Median *n* (%)	Range	*p* value
Age (y)	56	(37–74)	55	(43–81)	0.85
Body weight (kg)	58	(41–88)	62	(54–101)	0.17
BMI (kg/m^2^)	24	(18–37)	25	(22–33)	0.56
Male (hips)	6 (16%)		2 (29%)		
Indication (hips)					
Osteoarthritis due to DDH (Crowe grades I)	24 (65%)		2 (29%)		
Osteonecrosis	7 (19%)		3 (43%)		
Primary osteoarthritis	4 (11%)		2 (29%)		
Osteoarthritis due to trauma	1 (3%)				
Osteoarthritis due to osteochondritis dissecans	1 (3%)				
Follow-up period (y)	8	(7–9)	9	(9–9)	< 0.0001
Stem type (hips)					
Standard	3 (8%)				
High offset	34 (92%)		7 (100%)		
Head diameter (hips)					
28 mm	5 (14%)		7 (100%)		
32 mm	32 (86%)				
Head offset (hips)					
− 5 mm			1 (14%)		
+ 0 mm	17 (46%)		6 (86%)		
+ 3 mm	17 (46%)				
+ 6 mm	3 (8%)				
Unilateral THA (patients)	20 (63%)		4 (67%)		
Bilateral THA (patients)	5 (16%)		1 (17%)		
THA with other implants (patients)	7 (22%)		1 (17%)		
Flexural rigidity (Nm^2^)	45		82		

*BMI* body mass index; *DDH* developmental dysplasia of the hip; *THA* total hip arthroplasty

The stem was a proximal hydroxyapatite-coated, uncemented tapered wedge. It was composed of a titanium alloy (Ti-6Al-4 V) with a proximal diameter of 9 mm, a distal diameter of 10 mm, a contact length of 11.5 mm, and a trunnion taper angle of 5 degrees 34 min with a machined grooved imprinted surface, and two types of neck variations (standard and high-offset neck) were available.

Twenty patients had unilateral THA, 5 patients had bilateral THAs with the stem, and 7 patients had other metal implants including THA and total knee arthroplasty (Table 1).

### Surgical data

Surgery was performed through the posterior approach in the lateral decubitus position.

Before head impaction, the trunnion was carefully cleaned and dried. Full weight-bearing and walking were permitted on the first operative day. The acetabular component was an AHFIX Q3 shell (KYOCERA) composed of a titanium alloy. All patients received an Aquala liner (KYOCERA) that was highly cross-linked polyethylene grafted with poly(2-methacryloyloxyethyl phosphorylcholine) (PMPC) to reduce polyethylene wear and a CoCr head. PMPC is a methacrylate monomer with a phospholipid polar group that mimics the neutral phospholipids of biomembranes [11]. The head diameters were 28 and 32 mm in 5 and 32 hips, respectively, and the head offset was 0, 3, and 6 mm in 17, 17, and 3 hips, respectively (Table 1).

### Serum metal levels

Serum Co and Cr levels were examined twice, 1 year before final follow-up and at final follow-up, in all patients in the Kyoto Industrial Health Association (Kyoto, Japan), with detection levels of > 0.1 μg/L and > 0.2 μg/L for Co and Cr, respectively. One patient who had bilateral THAs had undergone unilateral revision surgery before the final blood examination. They were also examined once in 7 hips of 6 patients (1 patient had bilateral THAs, 1 patient had unilateral THA with other implants, and the others had unilateral THA) who underwent MoP THA with Synergy select II® (Ti-6Al-4 V alloy and 10/12 mm neck taper, taper angle of 5 degrees 40 min and taper length of 9.65 mm with a machined grooved imprinted surface) and a Reflection® cup (Smith & Nephew, Memphis, TN, USA) (Table 1). All patients received a CoCr 28-mm head, and they had no signs of aseptic loosening or periprosthetic osteolysis on CT at the final follow-up. We selected them as a control of serum metal concentrations because they had a larger trunnion diameter, had longer implantation time, and were composed of Ti-6A-4 V alloy.

### Flexural rigidity

Flexural rigidity of the trunnion was calculated following previously reported methods (13,14): Flexural rigidity = *E* × *I* = *E* × (π × (ND)^4^)/64).where *E* is the elastic modulus of the femoral neck alloy, *I* is the area moment of inertia of the trunnion cross section, and ND is the neck diameter. The elastic modulus was obtained from the literature [12]. NDz, ND at the geometric centroid, was calculated as: NDz = DD–Hz/H (DD–PD).where DD and PD represent the distal and proximal diameters, respectively, and *H* represents the height of the trunnion. Hz represents the location of the geometric centroid of the trunnion and was calculated as: Hz = H × (DD^2^ + 2DDPD + 3PD^2^)/4 (DD^2^ + DDPD + PD^2^).

### Clinical evaluation

Patients who received the 9/10 mm J-Taper stem underwent annual clinical evaluations with The Japanese Orthopaedic Association (JOA) hip score, which is composed of four parameters (pain, 40 points; range of motion, 20 points; walking ability, 20 points; and daily living activities, 20 points) preoperatively and postoperatively. The Hip Disability and Osteoarthritis Outcome (HOOS) score was also evaluated at the final follow-up. Both scores of revision cases were determined at the final follow-up before revision.

### Radiographic evaluation

CT was performed for all patients preoperatively and at the final follow-up. ZedHip software (LEXI, Tokyo, Japan) was used to evaluate cup and stem position precisely. Coronal stem alignment between the stem axis and the proximal femoral bone axis was calculated automatically, and positive values were defined as varus. Acetabular offset was defined as the perpendicular distance between the head center and the inner wall of the quadrilateral plate. Femoral offset was defined as the perpendicular distance between the head center and the stem shaft axis projected onto the coronal plane. Periprosthetic osteolysis was defined as a rounded or scalloped lesion around the implant at least 10 mm wide [13]. MRI was also performed on all but one patient at the final follow-up. Positive findings of abnormal tissue reactions were defined as an abnormal fluid collection or pseudotumor formation. Osteolysis on CT was evaluated by two senior authors, and positive findings on MRI were evaluated by an experienced radiologist unrelated to this study.

### Diagnosis of ALTR

ALTR was diagnosed in patients presenting with periprosthetic osteolysis > 10 mm on CT and a serum Co level > 1.0 μg/L twice, with one level > 1.6 μg/L or patients with abnormal tissue reactions including pseudotumor formation or large fluid collection with MARS MRI surrounding the hip with an elevated serum Co level > 1.0 μg/L [13, 14].

### Revision surgery and retrieval study

Revision surgery was performed in 4 hips of 3 patients through the posterior approach in the lateral decubitus position. At revision surgery, samples of collected fluid and tissue were obtained for bacterial culture and histopathological examination. Fretting and corrosion at the stem trunnion were evaluated using Goldberg’s classification [15] by two senior authors. Retrieved polyethylene liners and CoCr heads from 4 hips were rinsed with saline and sent to the Kyocera Research Center (Shiga, Japan). After ethylene oxide gas sterilization, the inner surface of an ex vivo liner was measured. Fretting and corrosion of the retrieved femoral head were evaluated using Goldberg’s classification with a digital microscope (KEYENCE, Osaka, Japan) and a scanning electron microscope (S-3400 N, HITACHI, Tokyo, Japan) by researchers in the Kyocera Research Center who were unrelated to this study.

### Statistics

Descriptive statistics were used to summarize the data. Individual serum Co and Cr levels were processed statistically using the average values of the two blood examinations. Intergroup differences in two (J-taper group and Synergy group) and in three groups (unilateral THA, bilateral THAs, and unilateral THA with other implant groups) of continuous variables were tested for significance using the Wilcoxon test and the Kruskal–Wallis test. Univariable linear regression analyses were performed to determine associations of two continuous outcomes (serum Co and Cr levels) and independent variables. *p* < 0.05 was considered significant.

## Results

### Serum Co and Cr levels

**Fig. 1:**
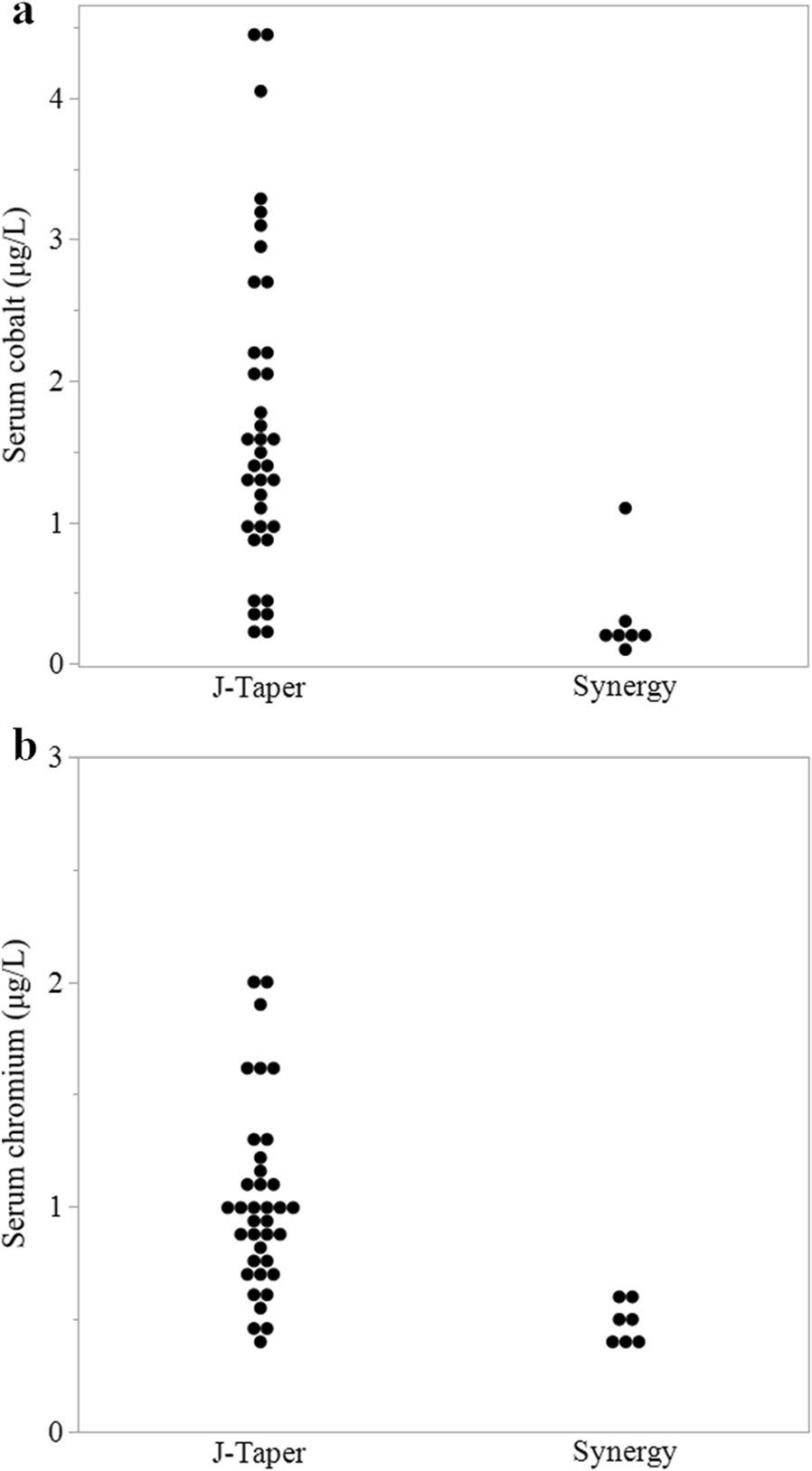
**a** Distribution of serum cobalt and **b** chromium levels in the J-Taper group and the Synergy group

**Fig. 2:**
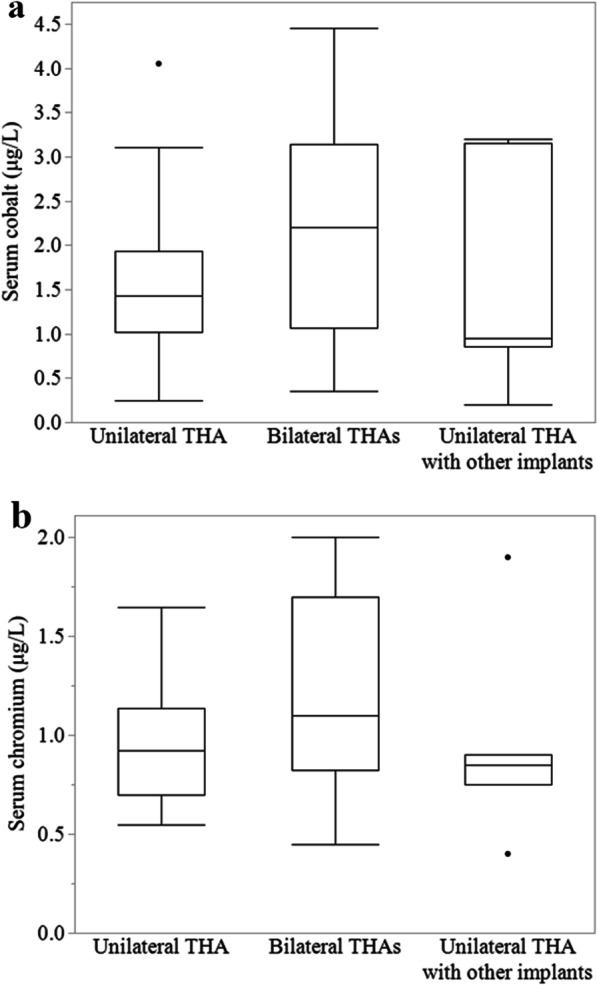
**a** Serum cobalt and **b** chromium levels in unilateral THA, bilateral THAs, and unilateral THA with other implants

All patients had detectable serum Co and Cr levels, with median values of 1.5 μg/L (range, 0.2 to 4.5) and 1.0 μg/L (range, 0.4–2.0) in the J-Taper group and 0.2 μg/L (range, 0.2–1.1) and 0.4 μg/L (range, 0.4–0.6) in the Synergy group, respectively. The serum metal levels were significantly higher in the J-Taper group than in the Synergy group (*p* = 0.0003 and *p* = 0.0003, respectively) (Fig. 1). There was no significant difference in serum Co and Cr levels among the unilateral THA, bilateral THAs, and unilateral THA with other implant groups in the J-Taper group (Fig. 2).

### Flexural rigidity

The flexural rigidity of the J-taper and Synergy stems was 45 Nm^2^ and 82 Nm^2^, respectively (Table 1).

### Postoperative radiographic assessment and ALTR

Osteolysis was confirmed in 7 patients (8 hips), and pseudotumor was detected in 3 patients (5 hips). ALTR was diagnosed in 5 hips of 3 patients.

### Factors associated with serum Co and Cr levels

Univariable regression analyses showed that the serum Co level increased with the postoperative JOA score of walking ability (coefficient = 0.17, 95% CI 0.01–0.3). The serum Cr level increased with weight (coefficient = 0.013, 95% CI 0.002–0.02) and the postoperative JOA score of walking ability (coefficient = 0.07, 95% CI 0.009–0.12). The serum Cr level also decreased with age (coefficient = − 0.02, 95% CI − 0.03–− 0.005) and HOOS symptoms (coefficient = − 0.06, 95% CI − 0.1–− 0.009) (Table 2).

**Table 2:** Factors associated with serum cobalt and chromium levels

	Serum cobalt (μg/L)		*p* value	Serum chromium (μg/L)		*p* value
	Coefficient	95% CI (range)		Coefficient	95% CI (range)	
Age	− 0.04	(− 0.08–0.006)	0.094	− 0.02	(− 0.03–− 0.005)	0.009
Weight	0.02	(− 0.01–0.05)	0.26	0.01	(0.002–0.02)	0.017
BMI	0.03	(− 0.07–0.12)	0.59	0.03	(− 0.003–0.06)	0.079
*Sex*						
Male (*n* = 6)	REF			REF		
Female (*n* = 31)	− 0.22	(− 0.74–0.3)	0.39	− 0.14	(− 0.3–0.04)	0.13
*Stem offset*						
Standard (*n* = 3)	REF			REF		
High offset (*n* = 34)	− 0.4	(− 1.1–0.3)	0.21	0.04	(− 0.2–0.3)	0.73
Head diameter						
28 mm (*n* = 5)	− 0.4	(− 0.9–0.2)	0.19	− 0.12	(− 0.3–0.09)	0.25
32 mm (*n* = 32)	REF			REF		
*Head offset*						
0 mm (*n* = 17)	0.5	(− 0.1–1.1)	0.097	0.2	(− 0.04–0.4)	0.11
3 mm (*n* = 17)	− 0.05	(− 0.7–0.6)	0.21	0.07	(− 0.15–0.29)	0.51
6 mm (*n* = 3)	REF			REF		
*Postoperative JOA score*						
Pain	0.005	(− 0.1–0.1)	0.92	− 0.014	(− 0.05–0.03)	0.47
Walk	0.17	(0.01–0.3)	0.0358	0.07	(0.009–0.12)	0.024
ROM	0.009	(− 0.2–0.2)	0.93	0.01	(− 0.07–0.09)	0.8
ADL	0.08	(− 0.08–0.2)	0.34	0.04	(− 0.02–0.09)	0.17
*HOOS score*						
Symptoms	− 0.08	(− 0.2–0.05)	0.22	− 0.06	(− 0.1–− 0.009)	0.019
Pain	− 0.009	(− 0.06–0.05)	0.76	− 0.015	(− 0.04–0.005)	0.14
Function in daily life	− 0.006	(− 0.03–0.02)	0.67	− 0.008	(− 0.02–0.002)	0.1
Function in sport and recreation	− 0.06	(− 0.2–0.04)	0.23	− 0.03	(− 0.07–0.001)	0.056
Hip-related QOL	0.03	(− 0.09–0.14)	0.62	− 0.012	(− 0.05–0.03)	0.55
Femoral offset	0.02	(− 0.05–0.08)	0.6	0.008	(− 0.014–0.03)	0.47
Acetabular offset	0.05	(− 0.08–0.18)	0.48	0.025	(− 0.02–0.07)	0.3
Cup anteversion	− 0.04	(− 0.1–0.02)	0.19	− 0.014	(− 0.04–0.01)	0.24
Cup inclination	0.03	(− 0.04–0.1)	0.33	0.02	(− 0.006–0.05)	0.12
Coronal stem alignment	0.02	(− 0.2–0.2)	0.88	0.004	(− 0.07–0.08)	0.9

*CI* confidence interval; *BMI* body mass index; *JOA* Japanese Orthopaedic Association Score; *ROM* range of motion; *ADL* activities of daily living; *HOOS* hip disability and osteoarthritis outcome score; *QOL* quality of life

### Revision and retrieval study

**Fig. 3:**
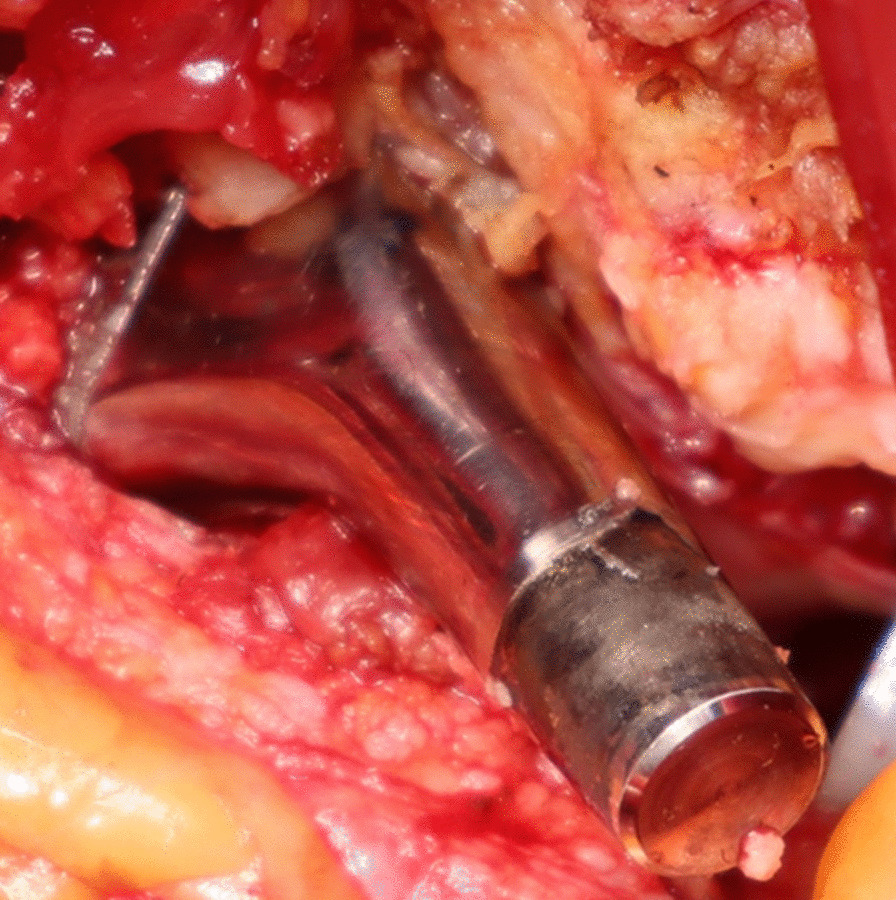
Corrosion of the trunnion at revision surgery. More than 10% of the trunnion surface contains black debris

**Fig. 4:**
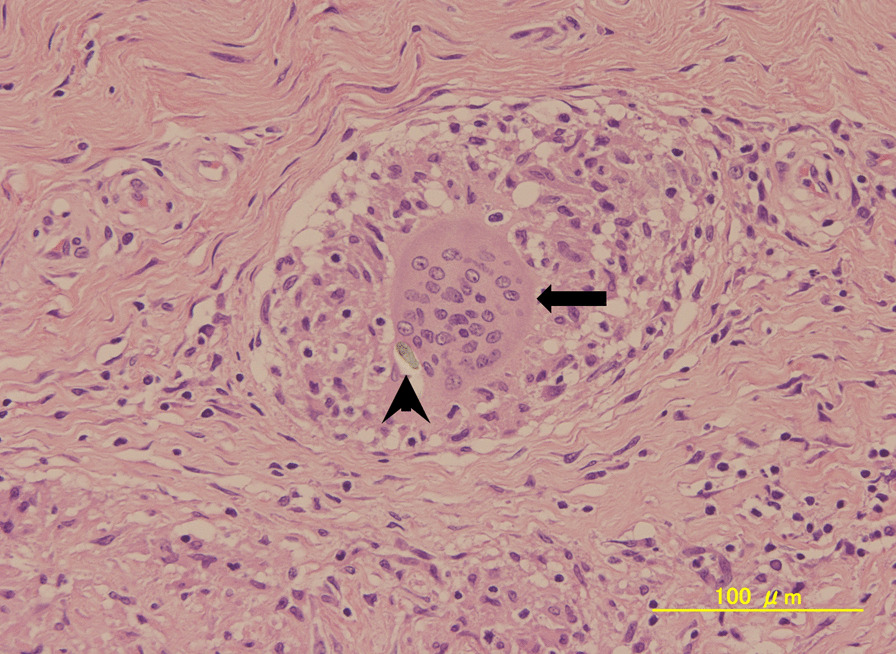
Histopathological examination of the periarticular tissue shows foreign material (black arrowheads) and foreign body giant cell reactions (black arrows)

**Fig. 5:**
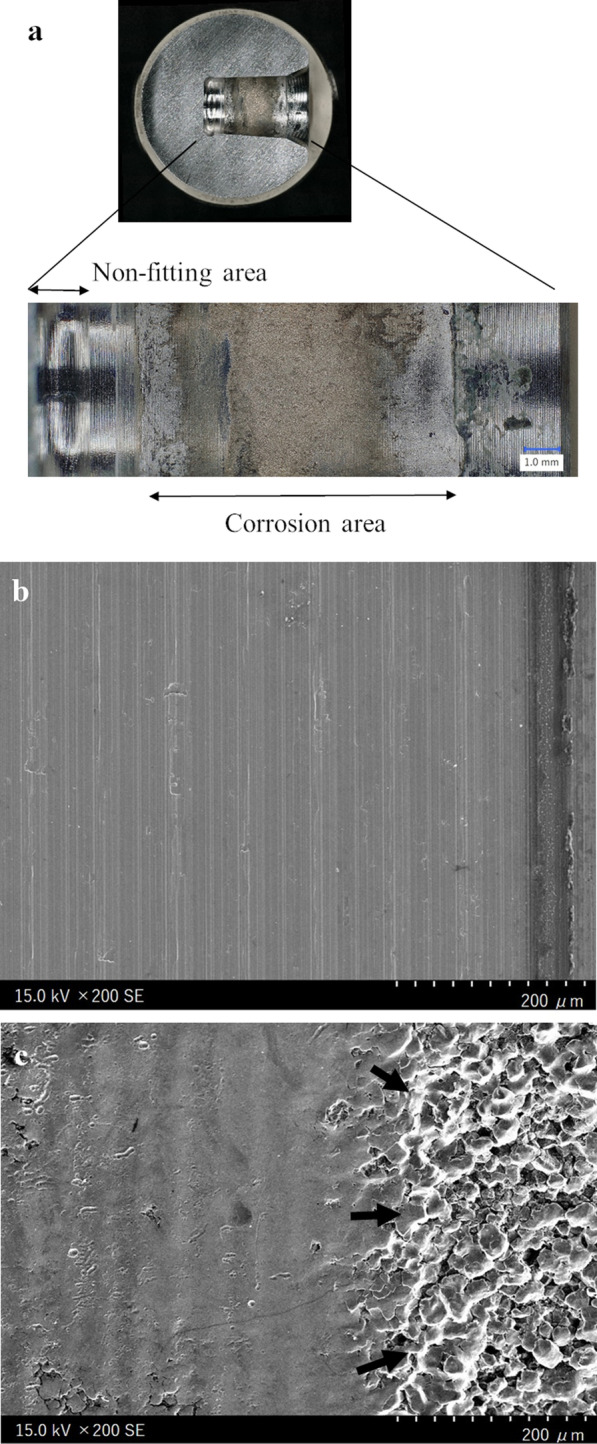
**a** Visualization of the retrieved femoral head by optical microscopy, and scanning electron microscopy images (200 × magnification) of **b** the non-fitting area and **c** intergranular corrosion (black arrows) in the corrosion area

Revision surgery using a ceramic head with a titanium alloy sleeve and liner exchange was performed in 4 hips of 3 patients, and 1 hip was awaiting revision surgery. All stem trunnions showed severe corrosion (Goldberg’s grade 4) (Fig. 3). No complications including dislocation, infection, and nerve palsy occurred after revision. All bacterial cultures were negative. Histopathological examination of the periarticular tissue in 2 hips showed necrotic tissues with foreign material and foreign body giant cell reactions (Fig. 4), and others showed only necrotic tissues. On examination of the retrieved femoral head, areas of corrosion were detected with optical and scanning electron microscopy (Fig. 5). All femoral heads showed severe corrosion (Goldberg’s grade 4). Abnormal polyethylene wear was not seen on the inner surface of an ex vivo liner.

## Discussion

The most important findings of this study were that a 9/10-mm trunnion stem with PMPC has high risks of metal release in MoP THAs in vivo, confirming the initial hypothesis.

### Limitations

The present study has several limitations. First, the sample size was small, which reduced the statistical power. Second, in the present study, only one type of stem from one manufacturer was used, and it is therefore difficult to generalize the results. Third, although serum metal concentrations were compared in two groups, the Synergy group may not be appropriate as the control. Almost all patients received a 32-mm head in the J-taper group, whereas all patients received a 28-mm head in the Synergy group. The implantation time of the Synergy group was also significantly longer. These differences can affect serum metal concentrations. Fourth, the liner was grafted with PMPC to reduce polyethylene wear [11], but its effect remained unclear in this study. Mouri et al. [16] reported the short-term clinical and radiographic results of metal-on-polyethylene grafted with PMPC THA. They found no periprosthetic osteolysis, and no revision surgery was performed in 3 years. Although Hosoi et al. reported that the PMPC graft disappeared from the bearing surface in the short term after THA in vivo [17], so far, there have been no reports of adverse complications of PMPC. Further studies will be needed to clarify its effect.

### Serum metal levels

Levin et al. reported that the mean serum Co and Cr concentrations were < 0.3 μg/L and 0.25 μg/L, respectively, in nine well-functioning, unilateral, metal-on-polyethylene THAs using a 14/16 or 12/14-mm trunnion stem with 10-year follow-up [18]. Martin et al. reported that mean serum Co and Cr levels were 0.51 μg/L and 0.39 μg/L, respectively, in 80 consecutive, well-functioning, unilateral, metal-on-polyethylene THAs using a 11/13, 12/14-mm, V40 or C-taper trunnion stem [19]. Compared to these previous reports, serum Co and Cr levels in the J-taper group were higher, and those in the Synergy group were comparable. Stem trunnions with smaller taper geometry showed less flexural rigidity [20], and it is known that less flexural rigidity is a risk factor for corrosion in retrieval studies [15]. In the present study, the 9/10-mm trunnion showed less flexural rigidity (45 Nm^2^), and all stem trunnions (*n* = 4) at revision surgery and retrieved femoral heads (*n* = 4) showed severe corrosion (Goldberg’s grade 4). Thinner stem trunnions can contribute to corrosion and metal release in vivo.

#### ALTR

Hussey et al. reported that the prevalence of ALTR was 3.2% (43 of 1352 hips) [3]. They used six types of stems with one type of taper (12/14) and found that one of them had a greater ALTR prevalence (4.7%, 36 of 769 hips). The present results showed a higher prevalence of ALTR (14%, 5 of 37 hips). Although we believed that metal release caused by the thinner stem trunnion with PMPC was one of the causes of the higher prevalence of ALTR, multiple factors, including the immune system, affect ALTR [21]. Further study will be needed to determine the cause of ALTR.

### Factors associated with serum Co and Cr levels

Previous clinical and retrieval studies have shown that head size [5], head offset [8], longer implantation time [11], varus stem position [22], and patient-related factors including weight [8] were risk factors for fretting and corrosion at the head–neck junction. Huot et al. also reported that patient activity level prior to the onset of symptoms showed a significant relationship to the corrosion severity at the head–neck junction with a modular stem in the retrieval study [23]. In the present study, both serum Co and Cr levels increased with the postoperative JOA score of walking ability. The serum Cr level also increased with weight and decreased with age. Other factors, including head size, head offset, varus stem position, weight, and acetabular and femoral offset were not significant. Increased mechanical loading at the head–neck junction in younger patients can contribute to fretting, corrosion, and metal release.

## Conclusion

A thinner trunnion with PMPC had higher risks of metal release and ALTR in primary MoP THA. Careful follow-up, serum metal measurement, and cross-sectional imaging are needed to detect ALTR and facilitate early revision.

## Data Availability

The datasets generated and/or analyzed during the current study are available from the corresponding author upon reasonable request.
